# Setting national nursing research priorities in Qatar: A Delphi survey

**DOI:** 10.1002/nop2.70010

**Published:** 2024-10-30

**Authors:** Albara Mohammad Ali Alomari, Kamaruddeen Mannethodi, Kalpana Singh, Jibin Kunjavara, George Vellaramcheril Joy, Ederlie Encarnacion Pitiquen, Badriya A. L. Lenjawi

**Affiliations:** ^1^ University of Doha for Science and Technology Doha Qatar; ^2^ Hamad Medical Corporation Doha Qatar

**Keywords:** Delphi survey, nurse, nursing administration, nursing education, nursing practice, nursing research, Qatar, research priority

## Abstract

**Aim:**

To establish consensus on the priorities of nursing research in Qatar regarding nursing administration, nursing practice and nursing education for the years 2023–2033.

**Design:**

Classic Delphi format over three phases.

**Methods:**

The first phase involved a qualitative round where participants identified 10 research priorities. In phase two, the collected sentences were analysed and grouped into themes. Participants were then requested to rate these sentences based on their perceived importance. During phase three, participants received their individual responses, the consolidated group response from the second round, and were given the opportunity to agree or disagree with the group ranking. The panel of experts consisted of 32 participants who agreed to participate in all three rounds. They included a mixture of nursing academics, clinical managers and nursing directors from public sectors and nursing schools in Qatar.

**Results:**

The experts reached a consensus on the research priorities for Qatar. These prioritized topics focused on improving patient care outcomes, enhancing workforce development, strengthening nursing leadership, integrating technology to improve nursing and nursing education and promoting evidence‐based practice.

**Conclusion:**

This research emphasizes the importance of progress in nursing to meet healthcare demands. Findings showed the need of future research to focus on improving nursing workforce and well‐being, leadership styles and technology, and evidence‐based practice and technology in nursing education.

**Impact:**

The impact of identifying nursing research priorities in Qatar will improve healthcare practices, fostering a culture of evidence‐based care. These advancements will motivate nurses to engage more actively in research, thereby enhancing overall healthcare outcomes.

**Public Contribution:**

Not applicable.

## 1 INTRODUCTION

Healthcare research needs more planning and strategic direction. In the past, research topics have mainly been developed by ‘enlightened individuals’ rather than by what is needed in the health services or what might be identified as the greatest problem areas by stakeholders (Alomari et al., 2020). Nurses in particular continuously face many new challenges, and it is important to identify and set research agendas to address these challenges (Haber & LoBiondo‐Wood, 2021).

For a research program to be considered clinically relevant, it is vital that researchers try to answer questions that are considered significant and meaningful by nurses (Alomari et al., 2022). As nursing research is recognized as a priority in many settings, the use of research findings that might provide effective and efficient nursing care is needed. However, without knowledge of the most pressing problems or questions affecting nursing research, efforts may be directed at areas that are not of the highest priority in today's healthcare systems. Setting a nursing research agenda at a national level will focus on the highest priority research problems, which may lead to more successful implementation of the research results (Tong et al., 2015). Setting nursing research agendas and identifying areas of research priority are strategies that have been reported in the United States (Yang et al., 2018) and Britain (Reay et al., 2014). No such study has been conducted in Qatar to identify nursing research priorities from different nursing stakeholders at a national level.

## 2 BACKGROUND

In Qatar, the healthcare sector is still overcoming ongoing challenges to improve patient outcomes. However, current data shows that 23% of Qataris and 18.3% of non‐Qataris suffer from Type 2 Diabetes Mellitus (T2DM). The country has the fifth highest obesity rate in the world (Syed et al., 2019). Additionally, 32.9% and 21.9% of the population suffer from high blood pressure and elevated cholesterol levels, respectively (Syed et al., 2020). These figures highlight the need to address chronic diseases as a significant public health issue in the country. In this challenging healthcare climate, identifying key areas of research in nursing is crucial to advance the profession and improve patient care. The focus of future research should be directed toward nursing management, practice and education, as key pillars essential to building a robust healthcare system and to be able to face future challenges (World Health Orgnisation, 2010). A growing body of research has showed how nursing management, education and practice impact quality of care and patient outcomes, and to better manage chronic conditions (Longhini et al., 2022).

The evolution of nursing research in Qatar is very recent; real scientific research work did not begin until the 2000s. However, a previous review of nursing research in Qatar between 2000 and 2015, found that only 57 studies were conducted by nurses, in collaboration with nurses or with nurse participants (Nashwan et al., 2017). Nowadays, building and sustaining nursing research is considered a priority for nursing leaders, academic bodies and the Ministry of Public Health (MoPH) in Qatar (Alomari et al., 2023). Efforts must be focused on research projects that are of the highest priority as perceived by nursing experts (Alomari et al., 2020). A previous Delphi survey was conducted in the Eastern Mediterranean region to explore the nursing research priorities (Sun et al., 2017). The study underscored the absence of consensus among the participating countries concerning certain priorities due to notable discrepancies between the participating countries. The authors recommended conducting research priority studies tailored specifically to each country (Sun et al., 2017).

Strengthening nursing and midwifery within the Middle East region is one of the five strategic priorities of the WHO (El Moubadder et al., 2023). Regional and country‐specific research is seen as a critical step (World Health Organization, 2021), and the identification of nursing education, practice and management priorities is imperative to ensure that the research conducted is efficient and relevant (Tong et al., 2015). As stated earlier, there is little evidence that identifying the research priorities in Qatar has been performed at the individual level while seeking consensus. There remains a gap in clear direction and consensus for clinical nursing research (McIlfatrick & Keeney, 2003).

## 3 THE STUDY

### 3.1 Aim

To establish consensus on the priorities of nursing research in Qatar on nursing administration, nursing practice, and nursing education in the years (2023–2033).

## 4 METHODS/METHODOLOGY

### 4.1 Design

The method used in this study was the ‘classic’ Delphi format. This means that each respondent is an expert in their area of interest (Skinner et al., 2015). The Delphi technique is a structured process that uses a series (or rounds) of questionnaires to gather information, and rounds are continued until a ‘group’ consensus is reached. A universally agreed proportion does not exist because the level used depends on sample numbers, research aim and resources. Therefore, the consensus for this study was at 65% or greater (McPherson et al., 2018).

#### 4.1.1 Study settings and recruitment

Three rounds of questionnaires were distributed to nurse leaders and academics to consolidate inputs aimed at a consensus on the priorities of nursing research in Qatar in the next 10 years on nursing administration, nursing practice and nursing education. Because the Delphi technique relied on engaging people who knew about a specific topic, purposive sampling was used. In this research, the panel comprised a combination of stakeholders and nursing experts. Experts have been defined as a group of ‘informed individuals’, or as ‘specialists’ in their field (Wilkes, 2015). The expert panel of this study included participants who are currently regarded as experts in nursing administration, nursing practice and nursing education in Qatar (Lopez, 2003). Three groups were identified, resulting in a total of 119 participants, including presidents of the Nursing Association of Qatar (*n* = 1), primary health care Nursing Executive (*n* = 1). Nursing executives, assistance executives and directors of nursing from different care levels who were currently working in the largest health care referral facilities in Qatar (*n* = 105), and Academics and deans of the only three schools of nursing in Qatar.

The potential participants were emailed the invitation and cover letter for the project. This step was taken to provide the potential participants with a comprehensive understanding of the Delphi process and to notify them that their commitment to participate would encompass multiple rounds of questionnaires and feedback spread across several months. The participants were requested to sign a consent form and send it back via email to the first author if they agreed to commit to the project. To ensure that participants who did not respond were followed up with, ongoing communication (via email and phone) were used every 2 weeks throughout this recruitment stage. The recruitment of experts and obtaining their approval took a total of 6 weeks. Once the experts agreed to take part in the study, they were added to the list of participants. Participants who did not respond by the end of the recruitment phase were considered uninterested in participating in the study and were not contacted further.

### 4.2 Data collection and analysis

The questionnaire consisted of two sections. The first section asked for demographic details. The second section consisted of the following three open‐ended questions that sought the participants' views on the top 10 most important research topics in Qatar for the next 10 years: (a) ‘What are the research priorities for nursing in Qatar in the next 10 years on nursing administration?’ (b) ‘What are the research priorities for nursing in Qatar in the next 10 years on nursing practice?’ and (c) ‘What are the research priorities for nursing in Qatar in the next 10 years on nursing education?’

The questionnaire was pilot tested with 10 professionals outside of nursing research (including five practical nurses, two health visitors, and three head nurses). These experts were presented with a list of questions regarding the survey design, layout, clarity of information and content. A 100% response rate of was achieved. In general, participants found the questionnaire to be well‐structured, clear and concise. They also stated that this process allowed them to identify topics that they considered relevant, which contributed to the content validity. Additionally, because respondents would be asked to rate the same benchmarks at least twice, this contributed to their reliability.

#### 4.2.1 Phase 1

The first round of this Delphi study was qualitative. The questionnaire in this phase was emailed to participants. The collected contents were compiled by two researchers and the suggested topics were grouped into domains (REDACTED). A reminder email was sent after 2 weeks. Phase 1 lasted for 1 month.

Data from phase 1 were content analysed by the first author (REDACTED). This analysis requires multiple steps. First, the data were assessed line by line, coded with temporary names, and then recoded until the categories became well‐defined. Second, the individual categories were examined to create meaningful relationships with other categories and subcategories. Finally, the researcher used precise criteria to develop themes to describe the data (Fram, 2013). The author created categories of data to make them easier to manage for panellists to respond to in subsequent phases using the constant comparative method (Fram, 2013). This analysis ensures that the statements are not repetitive and the meaning of the responses has not been changed. A total of 30 statements resulted in phase 1 with 10 statements under each domain (nursing practice, nursing education and nursing management) were sent to be ranked in phase two.

#### 4.2.2 Phase 2

The aim of this phase was to rank the most important research priority for nursing in Qatar. Participants were invited to rate research priorities on a 5‐point Likert scale, ranging from 1 to 5 (1 = unimportant, 2 = little important, 3 = moderately important, 4 = important and 5 = very important). An open‐ended question was included in the survey to allow experts to include other research questions that were not included in the survey. No additional statements have been provided in response to the open‐ended questions by the participants. After receiving the list, the overall group response from round two was collated and ranked based on the final score.

In phase two, questionnaires were imported into IBM SPSS Statistics (Version 27) for analysis. The research priorities statements were ranked based on the mean rating for each item on a 1–5 scale. For each research priority, the frequency of positive rankings of 3, 4 and 5 from the 1–5 scales were added together. The average of the aggregate scores of all three dimensions was used to define the priority regions and the average scores for each dimension were then used.

#### 4.2.3 Phase 3

Phase three of the Delphi was a quantitative round. In this phase, participants were given their own individual response from phase two, the overall group response from phase two, and a space to select agree or disagree with group ranking. If they disagreed, they were asked to provide a new ranking score on a scale of 1–5. The new ranked list was sent to all participants to inform them about the result and to have another opportunity to rank the list items again.

In phase three, frequencies of the agreement were added to determine the number of statements that reached consensus at this stage. The mean of each of these statements was calculated and used to rank the statements in order from most important to least important.

### 4.3 Ethical consideration

The study was approved by the REDACTED on 09th of March, 2023. The study was conducted in full compliance with the principles of the ‘Declaration of Helsinki’, Good Clinical Practice (GCP), and within the laws and regulations of MoPH in Qatar. All collected data were coded to maintain anonymity. The link between the code and the identifier was deleted at the end of the study. The participants were informed that their participation in this research would be voluntary. The potential for group bias was avoided because consensus is developed without interaction among participants.

## 5 FINDINGS

One hundred and nineteen participants were invited. Fourty participants consented to participate in the study with a total response rate of 33%. The majority of participants (65%) were female. Age ranges are mostly middle‐aged with the largest group between 45 and 54 years (43%), followed by that of 35–44 years (33%). Almost three‐quarters of participants (73%) had at least a master's degree in nursing and 15% had a PhD. Sixty‐five percent of the participants were directors of nursing, and 88% were from clinical settings. In phase 2, 32 participants responded. Eight participants did not return the surveys with a drop rate of 20%. Table 1 shows the sociodemographic details of the participants.

**TABLE 1:** Demographics of the participants.

Factor	Level	Value
N		40
Gender	Female	26 (65%)
	Male	14 (35%)
Age in years	35–44	13 (33%)
	45–54	17 (43%)
	55–64	7 (18%)
	65 and above	1 (3%)
	Not reported	2 (5%)
Current job title	Assistant executive director of nursing	6 (15%)
	Director of nursing	26 (65%)
	Executive director of nursing	3 (8%)
	Nursing academic	4 (10%)
	Not reported	1 (3%)
Department	Clinical/hospitals	35 (88%)
	Academic	2 (5%)
	Not reported	3 (8%)
Highest educational qualification	Master's degree	29 (73%)
	PhD	6 (15%)
	Missing	5 (13%)

The mean for nursing administration was 43.8 ± 3.4 with a range of (35, 50), the mean for nursing practice was 45.97 ± 5.95 with a range of (27, 55) and the mean for nursing education was 42.5 ± 5.98 with a range of (26, 50). The mean score for each theme with each statement is displayed in Table 2. In phase 2, a total of 28 topics (93%) were rated a mean of 4 or more (see Table 2). Three topics scored above 4.5 included the enhancement of the nursing workforce's development, well‐being and retention, leadership styles and management practices for a healthy work environment and creating care initiatives to enhance patient safety and optimize the overall patient experience.

**TABLE 2:** Ranking of research topics in domains based on highest mean in round 3.

Variables	Phase 2	Phase 3	*p*‐value
	Mean ± SD	Mean ± SD	
*N*	32	32	
*Nursing administration*			
The enhancement of the nursing workforce's development, well‐being and retention	4.9 ± 0.3	4.9 ± 0.3	0.999
Leadership styles and management practices for a healthy work environment	4.7 ± 0.5	4.7 ± 0.3	0.999
Create care initiatives to enhance patient safety and optimize the overall patient	4.6 ± 0.6	4.5 ± 0.4	0.405
The role of nursing administration on staff safety and job satisfaction	4.5 ± 0.9	4.4 ± 0.4	0.35
Developing nursing education and training programs to improve nurses' leadership	4.5 ± 0.6	4.2 ± 0.8	0.084
Exploring the Influence of organizational culture on the leadership practices of	4.3 ± 0.6	4.2 ± 0.5	0.446
Integration of technology and innovation in nursing administration	4.4 ± 0.8	4.2 ± 0.4	0.176
The Importance of interprofessional collaboration in nursing administration	4.4 ± 0.6	4.1 ± 0.7	0.059
Balancing financial considerations and economic value and patient care	3.9 ± 0.9	3.8 ± 0.5	0.555
The effects of policy and legislation on the role and responsibilities of nursing	3.9 ± 0.9	3.7 ± 0.6	0.267
*Nursing practice*			
Transforming nursing education to meet advances in healthcare technology and Evidence‐based practice	4.5 ± 0.8	4.5 ± 0.1	0.999
The integration of patient‐centered in nursing practices	4.4 ± 0.8	4.4 ± 0.4	0.999
Empowering nurses to practise to the full extent of their capabilities	4.4 ± 0.7	4.4 ± 0.3	0.999
Advancing nursing education and professional development.	4.4 ± 0.8	4.2 ± 0.5	0.203
Fostering teamwork and cooperation between nurses and other healthcare professional	4.2 ± 0.8	4.2 ± 0.2	0.999
Expanding the role and responsibilities of nurses to optimize healthcare outcome	4.2 ± 0.8	4.1 ± 0.6	0.549
Adopting a holistic approach to healthcare delivery and problem solving	4.3 ± 0.7	4.1 ± 0.6	0.199
Chronic disease management and palliative care	4.2 ± 1.0	4.1 ± 0.5	0.586
Embracing diversity and cultural differences in healthcare	4.1 ± 0.8	4.1 ± 0.2	0.999
Taking responsibility for improving healthcare on a global scale	4.0 ± 0.9	3.9 ± 0.6	0.578
Nursing education			
Promoting evidence‐based outcomes and research in nursing education	4.5 ± 0.8	4.5 ± 0.3	0.999
Integrate clinical‐based learning into nursing education programs	4.5 ± 0.7	4.5 ± 0.2	0.999
Utilizing technology‐based methods in nursing education	4.3 ± 0.9	4.3 ± 0.3	0.999
The value of nursing education programs on patient outcomes	4.3 ± 0.9	4.3 ± 0.3	0.999
The importance of family education for front‐line nurses	4.3 ± 1.0	4.2 ± 0.6	0.604
Strategies to enhance professional development and continuous learning among nursing	4.4 ± 0.7	4.2 ± 0.5	0.166
Cultural competence and diversity	4.2 ± 0.9	4.2 ± 0.2	0.999
Developing leadership skills and empowering nurse educators	4.3 ± 0.8	4.1 ± 0.6	0.233
Developing and evaluating nursing curricula and learning strategies	4.0 ± 0.9	3.9 ± 0.5	0.556
Examining the advantages and difficulties of interprofessional collaborations in	4.0 ± 1.0	3.9 ± 0.3	0.555

In phase three, 25 topics were ranked above 4 with groups with SD ranging from 0.3 to 0.8. Only two sentences scored a mean of more than 4.5 (The enhancement of the nursing workforce's development, well‐being and retention and leadership styles and management practices for a healthy work environment, Mean = 4.9 and 4.7 respectivly.

There were no statistical differences in the mean ranking of topics between phases 2 and 3 (see Table 2).

### 5.1 Consensus level

**FIGURE 1:**
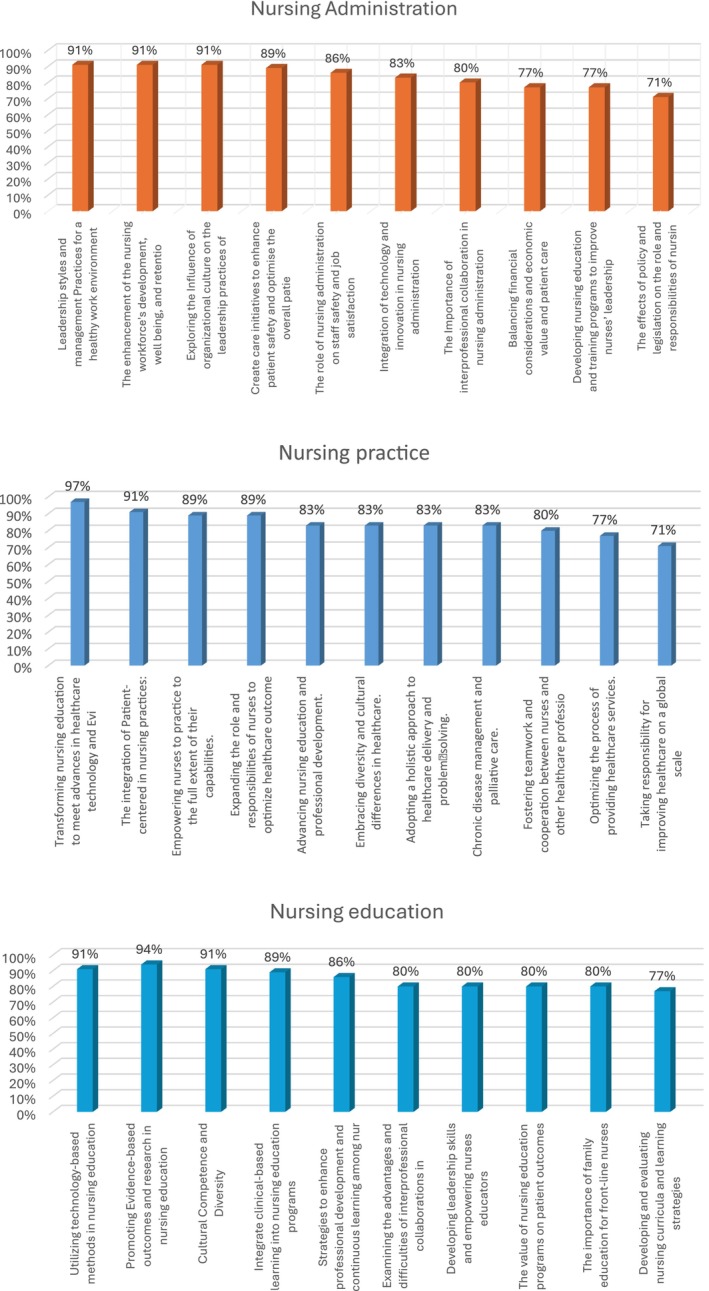
Agreement level in phase 3 for nursing administration, nursing practice and nursing education.

All research priority sentences achieved consensus (>65%). The lowest agreement percentage among all research priorities sentences was 71% (The effects of policy and legislation on the role and responsibilities of nursing, taking responsibility for improving health care on a global scale. The highest level of agreement (97%) was found in the nursing practice‐related category regarding the need to adapt nursing education to new developments in healthcare technology. Eight items were consistently ranked at or above 90% agreement. Figure 1 displays the percentage for all issues in the domains to indicate the level of agreement for phase 3

## 6 DISCUSSION

The current study aimed to identify nursing research priorities in the areas of nursing administration, nursing education and nursing practice for the coming decade in Qatar. These prioritized topics reflect the current and emerging needs of the Qatari healthcare system, with a particular focus on improving patient care outcomes, enhancing workforce development, strengthening nursing leadership, using and integrating technology to improve nursing and nursing education and promoting evidence‐based practice. These priorities are well aligned with the strategic objectives of the recent Qatar national health strategy to identify population and health system challenges. Under each domain of administration, education and practice, 10 research priorities were identified. This discussion of the findings focuses on the five top research areas and research problems.

Enhancement of the nursing workforce's development (quantity and quality), well‐being and retention were priorities identified by the leaders in the administration domain. This is not a surprising topic as Qatar, like many countries, suffered from nurse's retention and noticeable burnout after the COVID‐19 pandemic. Globally, the global health workforce experienced a shortage of workers due to the increased workload caused by the COVID‐19 pandemic (Alomari et al., 2023). This has led to calls for strategies to address the growing problem of job attrition in the health care sector (Poon et al., 2022). Consistent with previous studies, this result presents the concerns of nursing leaders in Qatar regarding a constant and ongoing issue surrounding nurse retention and developing strategies to improve their well‐being (Al‐Harahsheh et al., 2020). A number of studies conducted in Qatar have focused on factors that could affect nurses' retention rates, such as the work environment, job satisfaction, personal factors such as family and friends, the nurse–patient ratio and recognition and appreciation (Alameddine et al., 2017; El‐Jardali et al., 2013; Uddin, 2019). These studies recommended that more practical solutions to maintain a good workforce are still required in Qatar (Al‐Thawabiya et al., 2023). The present issues facing the nursing profession encompass staff shortages and turnover intention, which may have led participants in the current study to believe that workforce development and turnover are the top study priorities.

Leadership styles, organizational culture, and enhancing the skills and knowledge of leaders are identified as the second most crucial research priority by nursing experts in Qatar. This finding highlights the immense significance placed on these factors in the field of nursing. The prioritization suggests a shared understanding among nursing experts of the profound impact that leadership dynamics and elements of organizational culture can have on the overall quality of health care delivery and the well‐being of health care professionals. This research priority is in line with the existing literature highlighting the necessity for comprehensive studies to further investigate the consequences of diverse leadership styles and strategies within the particular cultural context of Qatar's health care system (Alomari et al., 2023). Research conducted on nursing leadership in Qatar indicates that to establish institutions capable of producing competent health managers and leaders, effective and adaptable nursing leadership styles are necessary (Al‐Thawabiya et al., 2023). However, nursing experts in this study believe that more research is needed to examine management policies that can enhance the capacity building of leaders and elucidate nursing management practices that foster a healthy and positive working environment.

In the nursing practice domain, priority is given to transforming nursing education to meet advances in health care technology. Given the rapid transformation of the health care industry, it is crucial to implement further innovations and improve procedures to meet the advancements in health care and achieve better outcomes (Kagan et al., 2023). The prioritization transforming nursing education under the nursing practice domain reflects the recognition within the health care community of the need to adapt educational frameworks to align with the rapid advances in health care technology. This emphasis shows a proactive response to the changing health care landscape, highlighting the importance of nursing professionals having contemporary knowledge and skills (Nashwan et al., 2023). The incorporation of technology in nursing education is perceived as an essential tactic, upheld by research that highlights the transformative potential of technologies such as virtual reality and information technologies in improving the quality, safety and overall effectiveness of nursing education (Sumpter et al., 2022). This strategic prioritization aligns with broader efforts to ensure that nursing education remains adaptable and responsive to the evolving demands and technological advancements in health care.

Considering the domain under education, the participants suggested more research on promoting evidence‐based outcomes and research in nursing education. Research education and integrating evidence to real clinical life is essential for establishing foundational research concepts and fostering a positive research attitude among individuals who are unfamiliar with research (Al‐Lenjawi et al., 2022). To meet higher professional requirements, nurses should have an opportunity to participate in ongoing education about the application of research evidence to clinical practice (Alomari et al., 2023). Evidence highlights that more studies are needed in research training programs that can enhance their appreciation for EBP and research, their interest in advanced education and meaningful dissemination of findings to improve patient care (Black et al., 2019).

The importance of technology in contemporary nursing education is further highlighted by the participants' suggestion to prioritize the integration of clinical‐based learning through advanced technologies, such as simulation. This emphasis on using simulation technologies agrees with previous literature that highlights their effectiveness in providing nurses with realistic clinical scenarios to enhance their decision‐making and practical skills (Kennedy et al., 2020). The participants' acknowledgement of these priority areas demonstrates a collective understanding of the importance of bridging the gap between theoretical knowledge acquisition and its practical application in clinical settings (Bayoumi et al., 2022). This deliberate focus aligns with studies that have emphasized the effectiveness of simulation‐based education in equipping nursing students with the necessary skills to tackle real‐world challenges, facilitating a smooth transition from educational settings to clinical practice (Foisy‐Doll, 2013).

The current study has limitations. Due to the reliance on the subjective evaluations of the expert panel in the Delphi method, possibly resulting in a lack of diverse viewpoints. Even though the study's design is comprehensive, it heavily relies on the consensus among a specific group of 32 participants, potentially not fully representing the wider nursing community in Qatar; for example, nurses from private sectors were excluded due to ethical constraints. Moreover, the classis Delphi method utilized here prioritizes controlled feedback and agreement over discussion, possibly suppress innovative concepts that do not align with the group's initial views. Nevertheless, participants were provided adequate time and freedom to contribute and provide feedback on the research priorities outlined.

## 7 CONCLUSION

This study highlighted the need of continuous progress in nursing practice, management and education to address the changing needs of the healthcare industry utilizing research. The study suggests a future where innovative teaching methods and practical experience will play a crucial role in shaping a resilient and skilled nursing workforce capable of addressing future global healthcare challenges. The results of this research highlighted the value of enhancing the growth, welfare and retention of nursing workforce. This emphasis is critical given the ongoing issues of staff turnover and the need for supportive work environments. The research priorities on leadership styles and the utilization of technology in nursing illustrates the constantly changing nature of healthcare systems and the need for leaders who can adeptly navigate challenges. Moreover, research priorities on evidence‐based practice and technology reflect a shared recognition of the importance of revolutionizing nursing education.

## AUTHOR CONTRIBUTIONS

Albara Alomari: Ethics application, data collection, data analysis, manuscript writing, editing. Kamaruddeen Mannethodi: Data analysis, manuscript writing. Kalpana Singh: Data analysis, manuscript writing. Jibin Kunjavara: Data collection, manuscript writing. George Vellaramcheril Joy: Data collection, manuscript writing. Ederlie E. Martinez: Ethics application, data collection, editing. Badriya AL Lenjawi: Manuscript writing, editing.

## FUNDING INFORMATION

The project was self‐funded.

## CONFLICT OF INTEREST STATEMENT

No conflict of interest to be declared.

## Data Availability

The data will be available from the corresponding author upon request.
